# Moment based gene set tests

**DOI:** 10.1186/s12859-015-0571-7

**Published:** 2015-04-28

**Authors:** Jessica L Larson, Art B Owen

**Affiliations:** 1 Department of Bioinformatics and Computational Biology, Genentech, Inc., South San Francisco, USA; 2 Currently at GenePeeks, Inc., Cambridge, USA; 3 Department of Statistics, Stanford University, Stanford, USA

**Keywords:** GSEA, Expression analysis, Permutation tests, ROAST

## Abstract

**Background:**

Permutation-based gene set tests are standard approaches for testing relationships between collections of related genes and an outcome of interest in high throughput expression analyses. Using *M* random permutations, one can attain *p*-values as small as 1/(*M*+1). When many gene sets are tested, we need smaller *p*-values, hence larger *M*, to achieve significance while accounting for the number of simultaneous tests being made. As a result, the number of permutations to be done rises along with the cost per permutation. To reduce this cost, we seek parametric approximations to the permutation distributions for gene set tests.

**Results:**

We study two gene set methods based on sums and sums of squared correlations. The statistics we study are among the best performers in the extensive simulation of 261 gene set methods by Ackermann and Strimmer in 2009. Our approach calculates exact relevant moments of these statistics and uses them to fit parametric distributions. The computational cost of our algorithm for the linear case is on the order of doing |*G*| permutations, where |*G*| is the number of genes in set *G*. For the quadratic statistics, the cost is on the order of |*G*|^2^ permutations which can still be orders of magnitude faster than plain permutation sampling. We applied the permutation approximation method to three public Parkinson’s Disease expression datasets and discovered enriched gene sets not previously discussed. We found that the moment-based gene set enrichment *p*-values closely approximate the permutation method *p*-values at a tiny fraction of their cost. They also gave nearly identical rankings to the gene sets being compared.

**Conclusions:**

We have developed a moment based approximation to linear and quadratic gene set test statistics’ permutation distribution. This allows approximate testing to be done orders of magnitude faster than one could do by sampling permutations.

We have implemented our method as a publicly available Bioconductor package, npGSEA (www.bioconductor.org).

**Electronic supplementary material:**

The online version of this article (doi:10.1186/s12859-015-0571-7) contains supplementary material, which is available to authorized users.

## Background

In a genome-wide expression study, researchers often compare the level of gene expression in thousands of genes between two treatment groups (e.g., disease, drug, phenotype, etc.). Many individual genes may trend toward differential expression, but will often fail to achieve significance. This could happen for a set of genes in a given pathway or system (a gene set). A number of significant and related genes taken together can provide strong evidence of an association between the corresponding gene set and treatment of interest. Gene set methods can improve power by looking for small, coordinated expression changes in a collection of related genes, rather than testing for large shifts in individual genes.

Additionally, single gene methods often require that all genes are independent of each other; this is not likely true in real biological systems. With known gene sets of interest, researchers can use existing biological knowledge to drive their analysis of genome-wide expression data, thereby increasing the interpretability of their results.

Mootha *et al.* [1] first introduced gene set enrichment analysis (GSEA) and calculated gene set *p*-values based on Kolmogorov-Smirnov statistics. Since then, there have been many methodological proposals for GSEA; no single one is always the best. For example, some tests are better for a large number of weakly associated genes, while others have better power for a small number of strongly associated genes [2].

One of the most important differences among gene set methods is the definition of the null hypothesis. Tian *et al.* [3] and Goeman and Bühlmann [4] (among others) introduce two null hypotheses that differentiate the general approaches for gene set methods. The first measures whether a gene set is more strongly related with the outcome of interest than a comparably sized gene set. Methods of this type typically rely on randomizing the gene labels to test what is often called the *competitive* null hypothesis. This is problematic because genes are inherently correlated (especially those within a set) and permuting them does not give a rigorous test [4].

The second type of approach is used to determine whether the genes within a set associate more strongly with the outcome of interest than they would by chance, had they been independent of the outcome. Methods that test this *self-contained* null hypothesis usually judge statistical significance by randomizing the phenotype with respect to expression data and assuming that gene sets are fixed. While we acknowledge that the *competitive* hypothesis is often of interest, we focus on methods that test the *self-contained* hypothesis in this paper.

Most current GSEA methods are based on random sampling of permutations. The initial GSEA [1] and widely used JG-score [5] methods both have closed form null distributions for their enrichment statistics, Kolmogorov-Smirnov and Gaussian, respectively, under appropriate assumptions. Both papers suggest permutation to gain robustness in case their assumptions don’t hold.

Lehmann and Romano [6] give a concise explanation of how permutation inference works. It is common to approximate the permutation distribution by a large Monte Carlo sample [7,8]. Monte Carlo permutation tests are simple to program and do not require parametric distributional assumptions. They also can be applied to almost any statistic we might wish to investigate. However, they are often computationally expensive, are subject to random inference, and fail to achieve continuous *p*-values. Each of these drawbacks is described in more depth below.

Testing many sets of genes becomes computationally expensive for two reasons. First, there are many test statistics to calculate in each permuted version of the data. Second, to allow for multiplicity adjustment, we require small nominal *p*-values to draw inferences about our sets, which in turn requires a large number of permutations. That is, to obtain a small adjusted *p*-value (e.g., via FDR, FWER, Bonferroni methods), one first needs a small enough raw *p*-value. In order to obtain small raw *p*-values, the number of permutations (*M*) must be large, thereby increasing computational cost. Suppose that a problem requires *p*-values as small as *ε*. Rules of thumb derived in our Discussion section show that one needs to take *M* between 3/*ε* and 19/*ε* to get adequate power.

Because permutations are based on a random shuffling of the data, we will usually obtain a different *p*-value for our set of interest each time we run our permutation analysis. That is, our inference is subject to a given random seed.

Permutations are subject to two granularity issues. As mentioned above, if we do *M* permutations, then the smallest possible *p*-value we can attain is 1/(*M*+1). We call this the *resampling granularity* problem.

There is also a *data granularity* problem. In an experiment with *n* observations, the smallest possible *p*-value is at least 1/*n*!. Sometimes the attainable minimum is much larger. For instance, when the target variable *Y* takes only the values 1 (*n*_1_ times) and 2 (*n*_2_ times) then the *p*-value cannot be smaller than $\epsilon = 1/{n_{1}+n_{2}\choose n_{1}}$. For instance, with *n*_1_=*n*_2_=5, we necessarily have *p*≥1/252. More generally, when *Y* has tied values, taking *K* distinct values *n*_*k*_ times each, the granularity is at least $\epsilon = \Pi _{k=1}^{K} n_{k}!/n!$. Rotation sampling methods such as ROAST are able to get around this data granularity problem [9], under a Gaussian assumption on the data. Increased Monte Carlo sampling with methods such as ROAST can mitigate the data granularity problem but not the resampling granularity problem.

Another aspect of the resampling granularity problem is that permutations give us no basis to distinguish between two gene sets that both have the same *p*-value 1/(*M*+1). There may be many such gene sets, and they may have meaningfully different effect sizes. Many current approaches address this problem by ranking significantly enriched gene sets by their corresponding test statistics. This practice only works if all test statistics have the same null distribution and correlation structure, which is not the case for many current GSEA methods. Additionally, the resulting broken ties do not have a *p*-value interpretation and cannot be directly used in multiple testing methods. To break ties in this way also requires the retention of both a *p*-value and a test statistic for inference, rather than just one value.

Because of each of these limitations of permutation testing, there is a need for an alternative to sampling permutations for gene set testing. The methods we present below are moment based approximations to the distribution of some gene set test statistics. We specifically target settings where there are no outliers, and where it is extremely expensive or even infeasible to do all possible permutations or to do the desired multiple of 1/*ε* permutations. In our view, that range starts where the number of distinct permutations is about 100,000, which corresponds to binary *Y* with about 10 observations in each group, or continuous *Y* with 9 or more values. If outliers are suspected, one could replace the genes by rank statistics. If the number of distinct permutations is much smaller than 100,000 then our software prints a warning. A small number of permutations could be exhaustively enumerated, and when the number is very small, then one would not expect a moment based approximation to be suitable.

Many different gene sets tests are possible when one combines all the choices that can be made. Recently, Ackermann and Strimmer [10] compared 261 different gene set tests, and found particularly good performance from a sum of squared single gene *t*-test statistics. There was also good performance for a plain sum of *t* statistics such as the JG-score [5]. These results were surprising because the winning test statistics are among the simplest that have been proposed. They note that the performance from the sum of squares is much better than the complicated GSEA method in [11]. In their simulations the excellent performance of those two classes of statistics extended also to statistics that merely summed correlation coefficients (or their squares). Those latter statistics are the ones that we use. We develop fast approximations to the permutation *p*-values for weighted sums and weighted sums of squares of correlation coefficients.

Our approximate *p*-values are not as computationally expensive, random, or granular as their permutation counterparts. Our proposal results in a single number on the *p*-value scale, suitable for use in multiple comparisons algorithms. We applied our approach to three public expression analyses. Our moment based *p*-values closely match those from an extensive permutation analysis. They also reveal disease-associated gene sets not previously discovered in these studies.

## Results

### The data

For definiteness, we present our notation using the language of gene expression experiments. Let *g*, *h*, *r*, and *s* denote individual genes and *G* be a set of genes. The cardinality of *G* is denoted |*G*|, or sometimes *p*. That is the same letter we use for *p*-value, but the usages are distinct enough that there should be no confusion. Our experiment has *n* subjects. The subjects may represent patients, cell cultures, or tissue samples.

The expression level for gene *g* in subject *i* is *X*_*gi*_, and *Y*_*i*_ is the target variable on subject *i*. *Y*_*i*_ is often a treatment, or a phenotype such as disease. We let *n*_*k*_ be the number of samples in the *k*th treatment group for *K* groups; $\Sigma _{k=1}^{K}n_{k}=n$. We center the variables so that

(1) $$ \sum\limits_{i=1}^{n}Y_{i} = \sum\limits_{i=1}^{n} X_{gi} = 0,\quad\forall g.  $$

The *X*_*gi*_ are not necessarily raw expression values, nor are they restricted to microarray values. In addition to the centering (1) they could have been scaled to have a given mean square. The scaling factor for *X*_*gi*_ might even depend on the sample variance for some genes *h*≠*g* if we thought that shrinking the variance for gene *j* towards the others would yield a more stable test statistic [12]. We might equally use a quantile transformation, replacing the *j* ^′^th largest of the raw *X*_*gi*_ by *Φ* ^−1^((*j*−1/2)/*n*) where *Φ* is the Gaussian cumulative distribution function. Further preprocessing may be advised to handle outliers in *X* or *Y*. We do require that the preprocessing of the *X*’s does not depend on the *Y*’s and vice versa.

### Test statistics

Our measure of association for gene *g* on our target variable is

(2) $$ \hat\beta_{g} = \frac1{n}\sum\limits_{i=1}^{n}X_{gi}Y_{i},  $$

the sample covariance of *X*_*gi*_ and *Y*_*i*_. If both *X*_*gi*_ and *Y*_*i*_ are centered and standardized to have variance 1, then $\hat \beta _{g}=\hat \rho _{g}$, the sample correlation between *Y* and gene *g*. The default in our software is to scale the *X*_*gi*_ values so that $\sum _{i=1}^{n} X_{\textit {gi}}^{2}=n$. With this default, our *p*-values are unaffected by scaling of *Y*_*i*_ and so they are equivalent to using the correlations.

If it often recommended to scale every gene to have unit variance, although the users may not always wish to. For instance in a setting where low expression values arise from probes with very low signal to noise level, scaling the genes may have the effect of inflating the noise in those probes relative to the signal in some others.

The usual *t*-statistic for testing a linear relationship between these variables is $t_{g} \equiv \sqrt {n-2}\hat \rho _{g}/(1-{\hat \rho _{g}^{2}})^{1/2}$. A Taylor approximation to fourth order yields

(3) $$ t_{g}\doteq \sqrt{n-2}\left(\hat\rho_{g}+\frac12{\hat\rho_{g}^{3}}\right)  $$

with an error of order ${\hat \rho _{g}^{5}}$. Gene-set tests are of most use when each individual $|\hat \rho _{g}|$ is small. In such cases *t*_*g*_ is very nearly a constant multiple of $\hat \rho _{g}$ and we expect permutation analyses using *t*-statistics to be very similar to those using correlations.

For reasons of power and interpretability, we apply gene set testing methods instead of just testing individual genes. Linear and quadratic test statistics have been found to be among the best performers for gene set enrichment analyses [10]; we thus consider two statistics for our approach:

$$\widehat{T}_{G,w} = \sum\limits_{g\in G}w_{g}\hat\beta_{g}\quad\text{and}\quad \widehat{C}_{G,w} = \sum\limits_{g\in G}w_{g}{\hat\beta_{g}^{2}}. $$

In this paper our null hypothesis is that *Y* is independent of (*X*_*g*_;*g*∈*G*). We test this null by formulating a statistic that is sensitive to the sort of departure we think is likely, as measured by either $\widehat {T}_{G,w}$ or $\widehat {C}_{G,w}$. If it were feasible, we would use the permutation distribution of the observed test statistic to get a *p*-value, but to save computation we develop moment approximations instead.

When all *w*_*g*_=1/|*G*|, then $\widehat {T}_{G,w}$ reduces to the average over *g*∈*G* of the correlation between *X*_*g*_, when the data are standardized. Such a test statistic will be sensitive to gene sets in which the non-null genes have correlations of the same sign with *Y*. If we have a prior expectation that some subset of *G* contains genes that move in opposite directions from the others in response to changes in *Y*, then we may choose positive *w*_*g*_ for those genes and negative *w*_*g*_ for the rest. Similarly if some subset of the genes in *G* are more important to the analyst, then those genes can be given larger absolute values of *w*_*g*_. The moment approximations work with general *w*_*g*_.

The statistic $\widehat {T}_{G,w}$ can approximate the JG score [5]. The JG score is

$$ \frac1{\sqrt{|G|}}\sum\limits_{g\in G}t_{g} \doteq\frac{\sqrt{n-2}}{\sqrt{|G|}}\sum\limits_{g\in G}\hat\rho_{g} =\frac{\sqrt{n-2}}{\sqrt{\text{sd}(Y)|G|}}\sum\limits_{g\in G}\frac1{\text{sd}(X_{g})}\hat\beta_{g} $$

where the approximation is good for small $\hat \rho _{g}$ and sd denotes standard deviation.

When *X*_*g*_ and *Y* are standardized then the statistics $\widehat {C}_{G}$ sums squared correlations. This statistics is useful when we expect that *Y* is associated with many of the genes *g*∈*G* but we do not know *a priori* what signs to expect for the correlations, nor even to expect that they mostly share the same sign.

The letters *T* and *C* are mnemonics for the *t* and *χ* ^2^ distributions that resemble the permutation distributions of these quantities. The *w*_*g*_ are scalar weights. For the quadratic statistics we will suppose that *w*_*g*_≥0. We won’t need this condition to find moments of *C*_*G*,*w*_. Any positive ${\hat \beta _{g}^{2}}$ contributes to evidence against the null hypothesis; negative weights would let strong evidence in one gene cancel evidence from another. Non-negative weights are also used to simplify our algorithm.

Although linear and quadratic test statistics are fairly restricted, they do allow customization through the weights *w*_*g*_, and they are very interpretable compared to more ad hoc statistics. They also performed well in [10] as we describe next.

### Motivation for these test statistics

Our chosen test statistics are supported by extensive simulations of Ackermann and Strimmer [10]. They compared 261 gene set testing methods. They consider per gene test statistics, that are then transformed and finally aggregated over the gene set, in various ways. Our quadratic test statistic $\widehat {C}_{G,w}$ is one of the ones that they particularly favor. The following notes are based on the summary in their pages 6–8.

They remark that they get roughly the same answers using a *t*-test, a moderated *t*-test, or a correlation, as the per gene statistic. Table two of their paper shows this. That was a surprising result because they had anticipated that moderated *t*-statistics might perform better. Moderated *t*-statistics use more stable estimates of the standard deviation of *X*_*gi*_, suitable for small samples. See [13,14] and [15] for moderation strategies. Ackermann and Strimmer [10] offer an explanation that the lack of benefit from moderation might be due to their simulation having sample sizes as large as 10. In our target setting, the sample sizes are on the order of 10 or more.

Our $\hat \beta _{g}$ is a sample correlation when, as usual, *X*_*gi*_ and *Y*_*i*_ are centered and scaled variables. They remark that squaring the per gene statistics is a ‘very useful transformation’. It works best on some of their scenarios. In the exceptional cases, untransformed quantities, like our linear test statistic, are best. They report that there is some advantage to a rank transformation prior to squaring. Such a transformation is possible in our framework, upon replacing *X*_*gi*_ by their ranks and then centering and scaling those ranks.

They found the mean or a maxmean over genes to be the best ways to combine the transformed statistics. We use a sum which gives the same *p*-values as using the mean. Medians or Wilcoxon statistics are better than the mean in one of their scenarios (correlated genes) for purposes of testing a competitive null. But that advantage vanishes when doing permutations as we do in testing the self-contained null, which is our focus here.

Finally, our linear statistic is motivated by trying to approximate the JG statistic, which is a sum of *t* statistics. Ackermann and Strimmer [10] found little difference between summing correlations and summing *t*-statistics, and our Taylor approximation above gives a reasonable explanation for their finding.

### Moment based reference distributions

When we permute the data, our sample statistics $\widehat {T}_{G,w}$ and $\widehat {C}_{G,w}$ take on new values, that we denote $\widetilde {T}_{G,w}$ and $\widetilde {C}_{G,w}$. To avoid the three main disadvantages to permutation-based analyses (cost, randomness, and granularity) discussed above, we approximate the distribution of the permuted test statistics $\widetilde {T}_{G,w}$ by Gaussians or by rescaled beta distributions. For quadratic statistics $\widetilde {C}_{G,w}$ we use a distribution of the form $\sigma ^{2}\chi ^{2}_{(\nu)}$ choosing *σ* ^2^ and *ν* to match the second and fourth moments of $\widetilde {C}_{G,w}$ under permutation. The family of scaled *χ* ^2^ distributions is the same as the family of gamma distributions.

For the Gaussian treatment of $\widetilde {T}_{G,w}$ we find $\sigma ^{2} = \text {var}\left (\widetilde {T}_{G,w}\right)$ under permutation using Eq. 8 of our Methods section and then report the *p*-value

$$p = \Pr\left(\mathcal{N}\left(0, \sigma^{2} \right) \le \widehat{T}_{G,w}\right), $$

where $\widehat {T}_{G,w}$ is the observed value of the linear statistic. The above is a left tail *p*-value. Two-tailed and right-tailed *p* values are analogous.

For the linear test statistic, a scaled beta distribution provides a useful alternative to the normal distribution. We use a scaled beta distribution, of the form *A*+(*B*−*A*)beta(*α*,*β*). It allows us to match four parameters of the permutation distribution (min, max, mean and variance) instead of just two as in the normal distribution. The beta(*α*,*β*) distribution has a continuous density function on 0<*x*<1 for *α*,*β*>0. We choose *A*, *B*, *α* and *β* by matching the upper and lower limits of $\widetilde {T}_{G,w}$, as well as its mean and variance. Using Eq. 8 from our Methods section we have

(4) $$\begin{array}{@{}rcl@{}} A & =& \min_{\pi} \frac1n\sum\limits_{i=1}^{n} \sum\limits_{g\in G}w_{g}X_{gi} Y_{\pi(i)},\\ B & =& \max_{\pi} \frac1n\sum\limits_{i=1}^{n} \sum\limits_{g\in G}w_{g}X_{gi} Y_{\pi(i)},\\ \alpha & =& \frac{A}{B-A}\left(\frac{AB}{\text{var}(\widetilde{T}_{G,w})}+1\right),\quad\text{and}\\ \beta & = &\frac{-B}{B-A}\left(\frac{AB}{\text{var}(\widetilde{T}_{G,w})}+1\right). \end{array} $$

The observed left-tailed *p*-value is

$$p = \Pr\left(\text{beta}(\alpha,\beta) \le \frac{\widehat T_{G,w}-A}{B-A}\right). $$

It is easy to find the permutations that maximize and minimize $\widetilde {T}_{G,w}$ by sorting the *X* and *Y* values appropriately as described in our Methods. The result has *A*<0<*B*. For the beta distribution to have valid parameters we must have *σ* ^2^<−*A* *B*. From the inequality of Bhatia and Davis [16], we know that *σ* ^2^≤−*A* *B*. There are in fact degenerate cases with *σ* ^2^=−*A* *B*, but in these cases $\widetilde {T}_{G,w}$ only takes one or two distinct values under permutation, and those cases are not of practical interest.

Like us, Zhou *et al.* [17] have used a beta distribution to approximate a permutation. They used the first 4 moments of a Pearson curve for their approach. Fitting by moments in the Pearson family, it is possible to get a beta distribution whose support set (*A*,*B*) does not even include the observed value $\widehat {T}_{G,w}$. That is, $\widehat {T}_{G,w}$ is even more extreme than it would have to be to get *p*=0; it is almost like getting *p*<0. We chose (*A*,*B*) based on the upper and lower limits of $\widetilde {T}_{G,w}$ to prevent our observed test statistic from falling outside the range of possible values of our reference distribution (Methods).

Our Beta approximation has the possibility of returning a *p*-value of 0 if the observed test statistic equals the most extreme possible value. A principled alternative that avoids returning 0 is to replace the left sided *p*_*L*_-value by

$$\widetilde{p}_{L}= \epsilon+(1-2\epsilon)p_{L} $$

where *ε* is the smallest possible permutation *p*-value. The corresponding right and central *p*-values are $\widetilde {p}_{R}=1-\widetilde {p}_{L}$ and $\widetilde {p}_{C}=2\min \left (\widetilde {p}_{L}, \widetilde {p}_{R}\right)$. When *X* has a continuous distribution and *Y* takes *K* distinct values *n*_1_,…,*n*_*K*_ times (due to ties) then the granularity is $\epsilon =\Pi _{k=1}^{K} n_{k}!/n!$.

For the quadratic test statistic $\widetilde {C}_{G,w}$ we use a $\sigma ^{2}\chi ^{2}_{(\nu)}$ reference distribution reporting the two-tailed *p*-value $\Pr \left (\sigma ^{2}\chi ^{2}_{(\nu)}\ge \widehat C_{G,w}\right)$ after matching the first and second moments of $\sigma ^{2}\chi ^{2}_{(\nu)}$ to $\mathbb {E}\left (\widetilde {C}_{G,w}\right)$ and $\mathbb {E}\left (\widetilde {C}_{G,w}^{2}\right)$ respectively. The parameter values are

$${} \nu = 2\frac{\mathbb{E}\left(\widetilde{C}_{G,w}\right)^{2}}{\text{var}\left(\widetilde{C}_{G,w}\right)} \quad\text{and}\quad \sigma^{2}=\frac{\mathbb{E}\left(\widetilde{C}_{G,w}\right)}{\nu} =\frac{\text{var}\left(\widetilde{C}_{G,w}\right)}{2\mathbb{E}\left(\widetilde{C}_{G,w}\right)}. $$

Our formulas for $\mathbb {E}\left (\widetilde {C}_{G,w}\right)$ and $\mathbb {E}\left (\widetilde {C}_{G,w}^{2}\right)$ under permutation are given in Eq. 5 of our Methods. Those formulas use $\mathbb {E}\left ({\widetilde {\beta }_{g}^{2}}\right)$ and $\text {cov}\left ({\widetilde {\beta }_{g}^{2}},{\widetilde {\beta }_{h}^{2}}\right)$ which we give in Corollaries 1 and 2 of our Methods.

Another alternative to permutations is rotation sampling. We have also shown in our Methods section that some of the moments of our test statistics are equal to rotation moments of those test statistics. The rotation-based values for $\mathbb {E}\left (\widetilde {T}_{G,w}\right)$, $\mathbb {E}\left (\widetilde {C}_{G,w}\right)$ and $\text {var}\left (\widetilde {T}_{G,w}\right)$ are same as for permutations; the variance of $\widetilde {C}_{G,w}$ is dependent upon the choice of rotation contrast matrix.

All of our reference distributions are continuous and the *χ* ^2^ and Gaussian ones are unbounded; hence they avoid the granularity problem of permutation testing. We have prepared a publicly available Bioconductor [18] package, npGSEA, which implements our algorithm and calculates the corresponding statistics discussed in this section.

### Parkinson’s Disease

We illustrate our method using publicly available data from three expression studies in Parkinson’s Disease (PD) patients (Table 1) [19-21]. All three experiments contain genome wide expression values measured via a microarray experiment. The values we use were normalized so that every gene had unit variance. PD is a common neurodegenerative disease; clinical symptoms often include rigidity, resting tremor and gait instability [22]. Pathologically, PD is characterized by neuronal-loss in the substantia nigra and the presence of *α*-synuclein protein aggregates in neurons [22].

**Table 1:** **Three data sets used for non-permutation GSEA**

**Reference**	**Tissue**	**# Affected**	**# Controls**
Moran	Substantia nigra	29	14
Zhang	Substantia nigra	18	11
Scherzer	Blood	47	21

#### Visualizing permutation distributions

**Figure 1:**
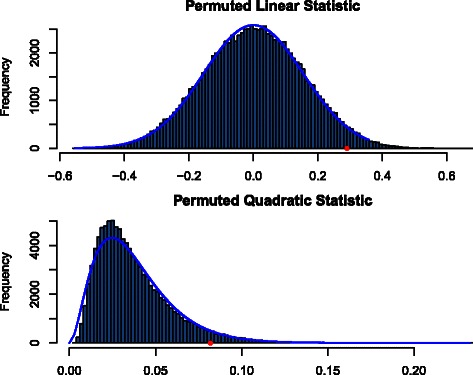
Distributions of permuted statistics resemble known probability densities. Top panel shows a permutation histogram for a linear test statistic for the steroid hormone signaling pathway gene set as described in the text. The bottom panel shows a quadratic test statistic. Solid red dots indicate the observed values and curves indicate parametric fits, based on normal and *χ* ^2^ distributions.

Using a selected set from the Broad Institute’s mSigDB v3.1 [23] and the presence of PD as a response variable from the Zhang *et al.* [20] dataset, we visualized both permutation distributions and our approximation of these distributions (Figure 1). As discussed above, we use a linear test statistic, $\widehat {T}_{G,w}=\sum _{g\in G}\hat \beta _{g}$, and a quadratic test statistic, $\widehat {C}_{G,w} = \sum _{g\in G}{\hat \beta _{g}^{2}}$, where $\hat \beta _{g}$ is a sample covariance between gene expression and, in this case, disease status. Figure 1 shows these two test statistics with a histogram of 99,999 recomputations of those statistics for permutations of treatment status versus gene expression for a steroid signaling pathway gene set from mSigDB. It is possible for histograms of permuted test statistics to be very complicated, but in practice, they often resemble familiar parametric distributions, as in Figure 1.

Using the fitted normal distribution to determine the rarity of the observed gene set statistic results in a two-tailed *p*-value of 0.0604 for the linear statistic while permutations yield *p*=0.0595. A fitted $\sigma ^{2}\chi ^{2}_{(\nu)}$ distribution results in *p*=0.0425 for the sum of squares gene set statistic, while permutations yield *p*=0.0458. The histogram for the sum of squared statistics has a somewhat sharper peak than its moment approximation. The *p*-values are nevertheless quite close; they are based on tail probabilities not the density itself.

#### Moment-based *p*-values tightly correlated with permutation *p*-values

We compared our non-permutation *p*-values to *p*-values for linear and quadratic statistics for the 6,303 gene sets from mSigDB’s curated gene sets and Gene Ontology (GO) [24] gene sets collections (v3.1). One gene set was removed because it contained only one gene in our experiments. The average size of these gene sets is 79.40 genes. For our gold standard we ran 999,999 permutations of the linear statistic and 499,999 permutations of the quadratic statistic. For all of our permutations, we first calculated the observed test statistic for each of the 6,303 gene sets and then permuted the *Y*_*i*_’s *M* times to obtain 6,303×*M* permuted test statistics. We next compared the pre-computed test statistic vector to our matrix of permuted test statistics.

**Figure 2:**
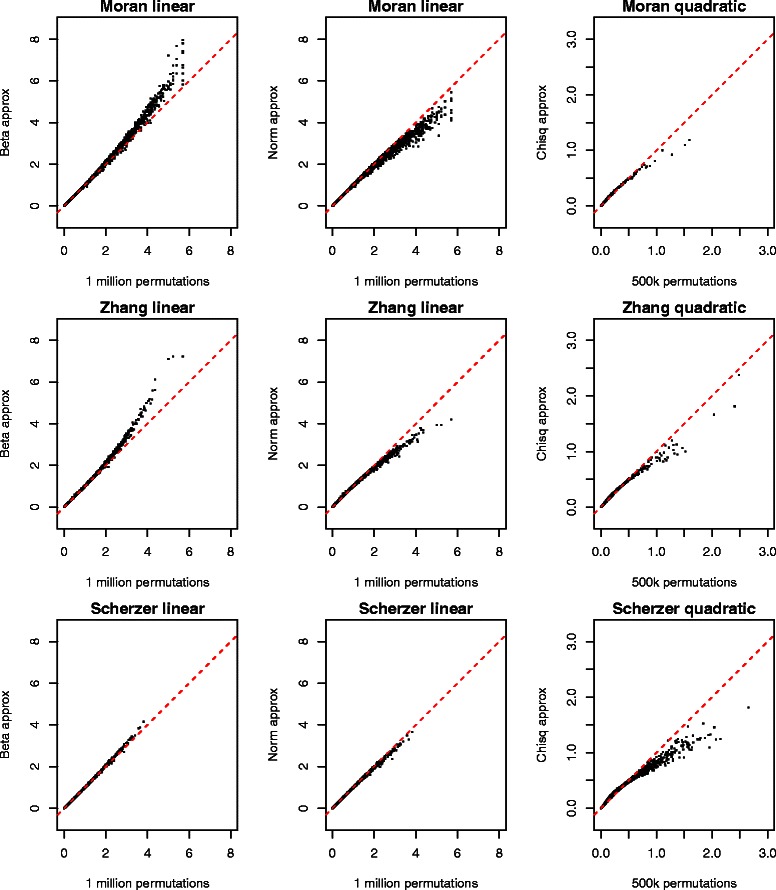
Permutation and moment-based *p*-values are tightly correlated. Permutation *p*-values (x-axis) versus moment-based *p*-values (y-axis) for 6,303 gene sets. The left two column represents results for a linear test statistic versus the beta and Gaussian approximations; the right-most column represents results for the sum of squares statistic versus the *χ* ^2^ approximation. Data come from three genome-wide expression studies. We applied the transformation − log10(*p*) to stretch the lower range of these distributions for a more informative visual. Red dotted lines represent the line *y*=*x*.

For each set, we computed left-sided *p*-values, *p*_*L*_, for the linear statistic and two-sided *p*-values, *p*_*Q*_, for the quadratic statistic using these permutations (Methods). We also computed the normal and beta approximations of *p*_*L*_ with our method. (Figure 2, left two panels). We converted these one-sided *p*-values to two-sided *p*-values via *p*=2 min(*p*_*L*_,1−*p*_*L*_). For very small *p*-values (<10^−3^), the beta and normal approximations sandwich the permutation values. At these values, the normal method is slightly conservative, while the beta approach is slightly anti-conservative. At larger *p*-values, the approximation-based values are almost identical to the permutation *p*-values.

The beta *p*-values can be quite a bit smaller than their permutation counterparts. Comparing two-tailed versions, we find that the beta approximate *p*-value is as much as 2.2-fold smaller for the Scherzer *et al.* [21] data set, 155-fold smaller for the Zhang *et al.* [20] data set, and almost 21,000-fold smaller for the Moran *et al.* [19] data set.

The very extreme ratio for the Moran data merits further investigation. It arose for a gene set in which the original data is more extreme than all 999,999 permuted versions. There were 16 gene sets where that happened. The sample of permutations does not distinguish among them; they all get a two-tailed *p*-value of 2×10^−6^. The smallest beta approximate *p*-value is about 10^−10^. To have sufficient power to verify such a *p*-value would require an extremely large number of permutations.

It is not too onerous to consider 16 tied gene sets. But a more reasonable number of permutations *M*=999 leads to 555 gene sets tied at the most significant possible level and even *M*=9999 leaves a tie among 186 of them.

For our quadratic test statistic, we fit our moment based $\sigma ^{2}\chi ^{2}_{(\nu)}$ approximation and computed two-sided *p*-values across all sets (Figure 2, right panel). We see that the smallest *χ* ^2^ non-permutation *p*-values are slightly conservative. This may reflect the boundedness of the permutation distribution combined with the unbounded right tail of the *χ* ^2^ distribution.

In each of the three experiments, there is a tight correlation between the permutation-based *p*-values of all sets and both of our moment-based methods (Table 2). Close rankings are important as one of the main tasks of gene set analysis is to order the gene sets so that followup investigations can be prioritized. The beta and normal approximations are almost identical. Our beta approximations are slightly closer to the gold standard than the normal approximations, but not by a practically important amount. The beta approximation has shorter tails than the Gaussian approximation. It yielded *p*-values somewhat smaller than permutations did, while the Gaussian approximation yielded *p*-values somewhat larger than the permutations did. The *χ* ^2^ approximations also reproduce the ranking of the gold standard quite well, though not as well as the normal and beta approximations to the linear statistic.

**Table 2:** **Spearman correlations between gold standard (999,999 and 499,999 permutations for linear and quadratic statistics) and approximation** ***p*** **-values**

**Reference**	**Normal** ***p***_***L***_	**Beta** ***p***_***L***_	**Normal** ***p***_***C***_	**Beta** ***p***_***C***_	**Chisq** ***p***_***Q***_
Moran	0.99991	0.99997	0.99973	0.99991	0.978
Zhang	0.99996	0.99997	0.99983	0.99991	0.990
Scherzer	0.99998	0.99999	0.99991	0.99997	0.994

*p*_L_ and *p*_*C*_ represent results for one and two-tailed linear test statistics, respectively. Chisq *p*_*Q*_ represents results for the sum of squares analysis.

#### Moment-based *p*-values are computationally inexpensive

For these data sets and 6,303 gene sets, both of the linear statistics, which have more or less the same rank-ordering of *p*-values as 999,999 permutations, could be approximated in about the amount of time it takes to compute 100 permutations (Table 3, top block). This is very close to our estimated cost of $|G|\doteq 80$ permutations. While this is a close match, we remark that the time to do *M* permutations is nearly an affine function *a*+*b* *M* with positive intercept *a*. At such small *M* the overhead costs dominated the total cost making the per permutation costs hard to resolve. The beta approximation was slightly slower than the Gaussian one because it involves the sorting of the data.

**Table 3:** **Time in seconds for** ***p*** **-value calculations for** ***6,303*** **gene sets in three genome-wide expression studies**

**Method**	**Moran**	**Zhang**	**Scherzer**
*M*=100	31.03	29.84	34.71
*M*=500	31.95	32.49	35.54
*M*=1,000,000	5010.17	4434.77	3933.15
Normal	29.74	27.00	34.66
Beta	30.79	31.88	37.89
*M*=30,000	9146.27	7217.59	11808.02
*M*=40,000	12256.54	9636.06	16545.60
*M*=50,000	16833.08	12564.06	21480.80
*M*=500,000	149588.37	129667.73	187067.91
*χ* ^2^	11020.62	10600.82	12677.15

Linear statistic results with *M* = 100, *M* = 500, and *M* = 1,000,000 permutations, and the normal and beta approximations are in the top block. Timings for the quadratic statistic with *M* = 30,000, *M* = 40,000, *M* = 50,000, and *M* = 500,000 permutations, and the *χ* ^2^ approximation are presented in the bottom block.

The *χ* ^2^ approximation to the quadratic statistic has a computational cost about as much as 35,000 to 45,000 permutations, yet has a similar rank-ordering of *p*-values from 499,999 permutations (Table 3, bottom block). For the quadratic statistic we expected our algorithm to cost as much as doing a number of permutations equal to a small multiple of the mean square gene set size. It cost about as much as 35,000 to 45,000 permutations while the mean square set size was 27,171.

#### Discovery of several gene sets associated with PD

After applying our permutation approximation methods to each dataset in 6,303 mSigDB gene sets, we found many significantly enriched gene sets, even after correcting for multiple testing with the Benjamini and Hochberg method [25] (two-sided adjusted *p*-value < 0.05). The most significantly enriched sets are associated with metabolism and mitochondrial function, neuronal transmitters and serotonin, epigenetic modifications, and the transcription factor FOXP3 (Additional file 1: Table S1). Each of these categories has some previously discovered association with PD, although not through traditional gene set methods (metabolism and mitochondrial function [22]; neuronal transmitters and serotonin [26]; epigenetic modifications [27]; FOXP3 [28]). Through our new gene set enrichment method, we discovered a relationship between the expression of these gene sets and PD.

## Discussion

Gene set methods are able to pool weak single gene signals over a set of genes to get a stronger inference. These methods and their corresponding permutation-based inferences are a staple of high throughput methods in genomics. Because an experiment for this purpose may have a few to hundreds of microarrays or RNA-seq samples, permutation can be computationally costly, and yet still result in granular *p*-values. In this paper, we introduce an approximate gene set method, which performs similarly to permutation methods, in a fraction of the computation time and which generates continuous *p*-values.

Permutation methods have some valuable properties that our approach does not share. Permutation inferences are exact at *p*-values that are a multiple of their underlying granularity. But typical modern gene set problems require finer resolution than permutation methods’ granularity allows, because of the large number of tests being made.

The second advantage of permutations is that they apply to arbitrarily complicated statistics. In our view, many of those complicated statistics are much harder to interpret and are less intuitive than the plain sum and sum of squared statistics we present. Others have observed that simple linear and squared statistics outperform more complex approaches [10]. Our method allows for the weighting of coefficients in our statistics, granting users access to additional useful and interpretable patterns.

Because of the disadvantages discussed above, there has long been interest in finding approximations to permutation tests. Eden and Yates [7] noticed that the permutation distribution closely matched a parametric distribution that one would get running an *F*-test on the same data. It has also been known since the 1940s that the permutation distribution of the linear test is asymptotically normal as *n* increases [29].

When a problem requires *p*-values as small as *ε* then a Monte Carlo approach requires a number of sample permutations in the range of 3/*ε* to 19/*ε*. The derivation is as follows. Suppose that we do *M*=*k*/*ε*−1 permutations. We can then claim a *p*-value of *ε* or smaller if *k*−1 or fewer sampled statistics exceed the observed value. With the true *p*-value (from enumeration) denoted by *p*, our power is then Pr(Bin(*M*,*p*)≤*k*−1). We suppose that the goal is to attain a *p*-value as small as *ε* with 80% power for *p* not much smaller than *ε*. For illustration, taking *ε*=10^−6^ with *p*=0.8*ε* and requiring power at least 80%, means that we require *k*≥19. The threshold is not sensitive to *ε*. The value *k*=19 is required for *ε*=10^−*r*^, *p*=0.8*ε* and integers *r*=2,3,…,40. If we only want 80% power in the event that *p*=0.5*ε*, then *k*=3 suffices.

It may easily happen that the necessary number *M*=*k*/*ε*−1 of permutations is onerous or even completely infeasible to do. In that case our moment based approximation provides a low cost substitute. The main limitation of our method is that we rely on a parametric approximation to the permutation distribution of our test statistic. An alternative is to employ a parametric model such as the Gaussian for *X*_*gi*_. Unfortunately, parametric models are also inexact due to lack of fit. This applies to ROAST [9] which assumes Gaussian data. The root of the problem is the non-existence of nonparametric confidence intervals for the mean [30]. In the case of npGSEA, one can do a spot check with a modest number, say *M*=10,000 permutations, to check on the accuracy of the moment based *p*-values.

Phipson and Smyth [31] remark that sampling permutations without replacement can be more efficient than independent sampling, and even allows access to *p*-values somewhat smaller than 1/(*M*+1) especially when the number of distinct permutation values is not very large. In our target settings though, the number of distinct permutation values becomes combinatorially large, and the bookkeeping to handle sampling without replacement is cumbersome.

Knijnenburg *et al.* [32] approach the granularity issue by taking a random sample of permutations and fitting a generalized extreme value (GEV) distribution to the tail of their distribution. They use several thousand permutations, and report better ordering of gene sets using their fits than using ordinary randomization. Knijnenburg *et al.* [32] report that the observed test statistic may be larger than the maximum of their fitted GEV distribution. They find that the problem is reduced (though perhaps not eliminated) by working with either the cube or the fifth power of the test statistic.

## Conclusions

We have developed a new and intuitive method for gene set enrichment analysis that is computationally inexpensive, and avoids the resampling granularity issue. A Gaussian, beta, or *χ* ^2^ approximation gives a principled way to break ties among genes or gene sets whose test statistics are larger than any seen in the *M* permutations. We applied our moment based approximations to three human Parkinson’s Disease data sets and discovered the enrichment of several gene sets in this disease, none of which were mentioned in the original publications.

## Methods

### Permutation procedure

A permutation of {1,2,…,*n*} is a reordering of {1,2,…,*n*}. There are *n*! permutations. We call *π* a *uniform random permutation* of {1,2,…,*n*} if it equals each distinct permutation with probability 1/*n*!.

In a permutation analysis, we replace *Y*_*i*_ by $\widetilde {Y}_{i}$ where $\widetilde {Y}_{i} = Y_{\pi (i)}$ for *i*=1,…,*n*. Then $\widetilde {\beta }_{g} = (1/n)\sum _{i=1}^{n}X_{\textit {gi}}\widetilde {Y}_{i}$, and when $\widetilde {Y}$ is substituted for *Y*, $\widehat {T}_{G,w}$ becomes $\widetilde {T}_{G,w}$ and $\widehat {C}_{G,w}$ becomes $\widetilde {C}_{G,w}$.

The *n*! different permutations form a reference distribution from which we can compute *p*-values. There are often so many possible permutations that we cannot calculate or use all of them. Instead, we independently sample uniform random permutations *M* times, getting statistics $\widetilde {C}_{m}=\widetilde {C}_{G,w,m}$, and similarly $\widetilde {T}_{m}$, for *m*=1,…,*M*. We then compute *p*-values by comparing our observed statistics to our permutation distribution:

$$ \begin{aligned} p_{Q} & = \frac{\#\left\{\widetilde{C}_{m}\ge \widehat{C}\right\}+1}{M+1} & p_{C} & = \frac{\#\left\{|\widetilde{T}_{m}|\ge |\widehat{T}|\right\}+1}{M+1}\\ p_{L} & = \frac{\#\left\{\widetilde {T}_{m}\le \widehat{T}\right\}+1}{M+1}, \quad\text{or} &p_{R} & = \frac{\#\left\{\widetilde{T}_{m}\ge \widehat{T}\right\}+1}{M+1}, \end{aligned} $$

where *p*_*Q*_ and *p*_*C*_ are *p*-values for two-sided inferences on the quadratic and linear statistic, respectively, and *p*_*L*_ (left) and *p*_*R*_ (right) are for one-sided inferences based on the linear statistic. We use the mnemonic *C* in *p*_*C*_ to denote the central (or two-sided) *p*-value, which corresponds to a central confidence interval. The +1 in numerator and denominator of the *p*-values corresponds to counting the sample test statistic as one of the permutations. That is, we automatically include an identity permutation. After adding 1, the permutation distribution of the *p*-value is uniform on {1/(*M*+1),2/(*M*+1),…,1}.

### Permutation moments of test statistics

Under permutation, $\mathbb {E}\left (\widetilde {Y}_{i}\right)=0$ by symmetry, and so $\mathbb {E}\left (\widetilde {\beta }_{g}\right)=0$ too. We easily find that,

(5) $$\begin{array}{@{}rcl@{}} \mathbb{E}\left(\widetilde{T}_{G,w}\right) & =& 0,\\ \text{var}\left(\widetilde{T}_{G,w}\right) & = &\sum\limits_{g\in G}\sum\limits_{h\in G} w_{g}w_{h}\text{cov}\left(\widetilde{\beta}_{g},\widetilde{\beta}_{h}\right)\\ \mathbb{E}\left(\widetilde{C}_{G,w}\right)&=& \sum\limits_{g\in G}w_{g}\mathbb{E}\left({\widetilde{\beta}_{g}^{2}}\right),\quad\text{and}\\ \text{var}\left(\widetilde{C}_{G,w}\right) &=& \sum\limits_{g\in G}\sum\limits_{h\in G} w_{g}w_{h}\text{cov}\left({\widetilde{\beta}^{2}_{g}},{\widetilde{\beta}^{2}_{h}}\right). \end{array} $$

The means, variances and covariances in (5) are taken with respect to the random permutations with the data *X* and *Y* held fixed. We adopt the convention that moments of permuted quantities are taken with respect to the permutation and are conditional on the *X*’s and *Y*’s. This avoids cumbersome expressions like $\mathbb {E}\!\left (\!{\widetilde {\beta }^{2}_{g}}\mid X_{\textit {gi}}, Y_{i}, g\in G\!\right)$.

We will need the following even moments of *X* and *Y*:

$$\begin{array}{@{}rcl@{}} \mu_{2} & =& \frac1n\sum\limits_{i=1}^{n} {Y_{i}^{2}},\quad \mu_{4} = \frac1n\sum\limits_{i=1}^{n} {Y_{i}^{4}},\\ \bar X_{gh} &=& \frac1n\sum\limits_{i=1}^{n} X_{gi}X_{hi},\quad\text{and}\\ \bar X_{ghrs} & =& \frac1n\sum\limits_{i=1}^{n} X_{gi}X_{hi}X_{ri}X_{si} \end{array} $$

for *g*,*h*,*r*,*s*∈*G*. Although our derivations involve *O*(*p* ^4^) different moments when the gene set *G* has *p* genes, our computations do not require all of those moments.

#### **Lemma1**.

For an experiment with *n*≥2 including genes *g* and *h*,

$$\mathbb{E}\left(\widetilde{\beta}_{g}\widetilde{\beta}_{h}\right) = \frac{\mu_{2}\bar X_{gh}}{n-1}. $$

#### *Proof*.

This appears in [33] but we prove it here to keep the paper self-contained. First

$$n^{2}\mathbb{E}\left(\widetilde{\beta}_{g}\widetilde{\beta}_{h}\right) = \sum\limits_{i}\sum\limits_{i'}X_{gi}X_{hi'}\mathbb{E}\left(\widetilde{Y}_{i}\widetilde{Y}_{i'}\right) $$

Recall that $\mu _{2} =\frac 1n\sum _{i=1}^{n} {Y_{i}^{2}}$. Then

$$\mathbb{E}\left(\widetilde{Y}_{i}\widetilde{Y}_{i'}\right) =\left\{ \begin{array}{ll} \mu_{2},& i'=i\\ -\dfrac1{n-1}\mu_{2}, & i'\ne i \end{array} \right. $$

and so

$$\begin{array}{@{}rcl@{}} n^{2}\mathbb{E}\left(\widetilde{\beta}_{g}\widetilde{\beta}_{h}\right) &=& \sum\limits_{i}\sum\limits_{i'}X_{gi}X_{hi'}\mathbb{E}\left(\widetilde{Y}_{i}\widetilde{Y}_{i'}\right)\\ & =& \mu_{2}\sum\limits_{i}\sum\limits_{i'}X_{gi}X_{hi'} \left(1_{i=i'} - \frac1{n-1}1_{i\ne i'}\right)\\ & =& \mu_{2}\sum\limits_{i}\sum\limits_{i'}X_{gi}X_{hi'} \left(\frac{n}{n-1}1_{i=i'} - \frac1{n-1}\right)\\ & = &\frac{n}{n-1}\mu_{2}\sum\limits_{i}X_{gi}X_{hi}\\ &\equiv& \frac{n^{2}}{n-1}\mu_{2}\bar X_{gh}, \end{array} $$

proving Lemma 1.

#### **Corollary1**.

For an experiment with *n*≥2 including genes *g* and *h*,

$$\text{cov}\left(\widetilde{\beta}_{g},\widetilde{\beta}_{h}\right)=\mu_{2}\bar{X}_{gh}/(n-1). $$

#### *Proof*.

This follows from Lemma 1 because $\mathbb {E}\left (\widetilde {\beta }_{g}\right)=0$.

From Corollary 1, we see that the correlation between permuted test statistics $\widetilde {\beta }_{g}$ and $\widetilde {\beta }_{h}$ is simply the correlation between expression values for genes *g* and *h*.

#### **Lemma2**.

For an experiment with *n*≥4 including genes *g*,*h*,*r*,*s*,

$$\mathbb{E}\left(\widetilde{\beta}_{g}\widetilde{\beta}_{h}\widetilde{\beta}_{r}\widetilde{\beta}_{s}\right) =\left(\begin{array}{l} {\mu_{2}^{2}}\\ \mu_{4} \end{array} \right)^{\mathsf{T}} A^{\mathsf{T}} B\left(\begin{array}{l} \bar{X}^{*}_{ghrs}/n^{2}\\[1ex] \bar{X}_{ghrs}/n^{3} \end{array} \right) $$

where $\bar {X}^{*}_{\textit {ghrs}} =\bar {X}_{\textit {gh}}\bar {X}_{\textit {rs}}+\bar {X}_{\textit {gs}}\bar {X}_{\textit {hr}}+\bar {X}_{\textit {gr}}\bar {X}_{\textit {hs}}$, with *A* ^T^ given by

$$\left(\begin{array}{lcccc} 0 & 0 & \frac{n}{n-1} & \frac{-n}{(n-1)(n-2)} & \frac{3n}{(n-1)(n-2)(n-3)}\\[2ex] 1 & \frac{-1}{n-1} & \frac{-1}{n-1} & \frac2{(n-1)(n-2)} & \frac{-6}{(n-1)(n-2)(n-3)} \end{array} \right), $$

and

$$B =\left(\begin{array}{rr} 0 & 1\\ 0 & -4\\ 1 & -3\\ -2 & 12\\ 1 & -6 \end{array} \right). $$

#### *Proof*.

The fourth moment contains terms of the form

$$X_{gi}X_{hj}X_{rk}X_{\textit{s}\ell}\mathbb{E}\left(\,\widetilde{Y}_{i}\widetilde{Y}_{j}\widetilde{Y}_{k}\widetilde {Y}_{\ell}\right) $$

and there are different special cases depending on which pairs of indices among *i*, *j*, *k* and *ℓ* are equal. We need the following fourth moments of *Y* in which all indices are distinct:

$$\begin{array}{@{}rcl@{}} \mu_{4k} &= &\mathbb{E}\left(\widetilde{Y}_{i}^{4}\right)\\ \mu_{3k} & =& \mathbb{E}\left(\widetilde{Y}_{i}^{3}\widetilde{Y}_{j}\right)\\ \mu_{2p} & =& \mathbb{E}\left(\widetilde{Y}_{i}^{2}\widetilde{Y}_{j}^{2}\right)\\ \mu_{1p} & =& \mathbb{E}\left(\widetilde{Y}_{i}^{2}\widetilde{Y}_{j}\widetilde{Y}_{k}\right)\\ \mu_{\emptyset} & =& \mathbb{E}\left(\widetilde Y_{i}\widetilde{Y}_{j}\widetilde{Y}_{k}\widetilde{Y}_{\ell}\right), \end{array} $$

and where the subscripts are mnemonics for terms four of a kind, three of a kind, two pair, one pair and nothing special.

We can express all of these moments in terms of *μ*_2_ and $\mu _{4} = (1/n)\sum _{i=1}^{n}{Y_{i}^{4}}$. Each moment is a normalized sum over distinct indices. We can write these in terms of normalized sums over all indices. Many of those terms vanish because $\sum _{i}Y_{i}=0$.

Let $\sum ^{*}$ represent summation over distinct indices, as in

$$\begin{array}{@{}rcl@{}} \underset{ij}\sum^{*} f_{ij} &=& \sum_{i=1}^{n}\sum\limits_{j=1,j\ne i}^{n} f_{ij},\\ \underset{ijk}\sum^{*} f_{ijk}& = &\sum\limits_{i=1}^{n}\sum\limits_{j=1,j\ne i}^{n} \sum\limits_{k=1,k\ne i,k\ne j} f_{ijk} \end{array} $$

and so on. We can write these sums in terms of unrestricted sums:

$$\begin{array}{@{}rcl@{}} \underset{ij}\sum^{*} f_{ij} & =& \sum\limits_{ij}f_{ij} - \sum\limits_{i} f_{ii}\\ \underset{ijk}\sum^{*} f_{ijk} & =& \sum\limits_{ijk}f_{ijk} - \sum\limits_{ij}\left(\,f_{iij}+f_{iji}+f_{ijj}\right) + 2\sum\limits_{i}f_{iii},\quad\text{and}\\ \underset{ijk\ell}\sum^{*} f_{\textit{ijk}\ell} & = &\sum_{\textit{ijk}\ell}f_{\textit{ijk}\ell}\,-\,\sum_{ijk}\left(\, f_{ijki}\! +\! f_{ijkj} \,+\, f_{ijkk} \,+\, f_{ijik} \!+ \! f_{ijjk} \,+\, f_{iijk} \right)\\ && +\!\sum\limits_{ij}\!\left(2\left(f_{ijjj} + f_{ijii} \,+\, f_{iiji} + f_{iiij}\right) \,+\, f_{ijij} + f_{ijji} + f_{iijj} \right)\\ &&- 6\sum\limits_{i} f_{iiii}. \end{array} $$

See Gleich and Owen [34] for details.

We will use the last expression in a context where *f*_*ijkℓ*_ vanishes when summed over the entire range of any one of its indices. In that case

(6) $$\begin{array}{@{}rcl@{}} \underset{ijk\ell}\sum^{*} f_{\textit{ijk}\ell} & =& \sum\limits_{ij}\left(\, f_{ijij} + f_{ijji} + f_{iijj} \right)- 6\sum\limits_{i} f_{iiii}. \end{array} $$

We also use the notation *n* ^(*k*)^=*n*(*n*−1)(*n*−2)⋯(*n*−*k*+1), often called ‘*n* to *k* factors’, where *k* is a positive integer. Now

$$\begin{array}{@{}rcl@{}} \mu_{4k} &=& \frac1n\sum\limits_{i=1}^{n}{Y_{i}^{4}} = \mu_{4},\\ \mu_{3k} & =& \frac1{n^{(2)}} \underset{ij}\sum^{*} {Y_{i}^{3}}Y_{j} = \frac1{n^{(2)}} \left(\sum\limits_{ij} {Y_{i}^{3}}Y_{j} -\sum\limits_{i}{Y_{i}^{4}}\right)\\ & =& -\frac{\mu_{4}}{n-1},\\ \mu_{2p} & = &\frac1{n^{(2)}}\underset{ij}\sum^{*} {Y_{i}^{2}}{Y_{j}^{2}} = \frac1{n^{(2)}}\left(\sum\limits_{ij}{Y_{i}^{2}}{Y_{j}^{2}} -\sum\limits_{i} {Y_{i}^{4}}\right)\\ &= &\frac1{n-1}\left(n{\mu_{2}^{2}}-\mu_{4}\right),\quad\text{and}\\ \mu_{1p} &=& \frac1{n^{(3)}}\underset{ijk}\sum^{*}{Y_{i}^{2}}Y_{j}Y_{k}\\ &=& \frac1{n^{(3)}}\!\!\left(\sum\limits_{ijk} {Y_{i}^{2}}Y_{j}Y_{k} \,-\,\sum_{ij}\!\left(2{Y_{i}^{3}}Y_{j} \,+\, {Y_{i}^{2}}{Y_{j}^{2}}\right) \,+\,2\sum\limits_{i}{Y_{i}^{4}} \!\right)\\ & =& \frac{-n{\mu_{2}^{2}}+2\mu_{4}}{(n-1)(n-2)}. \end{array} $$

Finally using (6), *n* ^(4)^ *μ*_∅_ equals

$$\begin{array}{@{}rcl@{}} \underset{ijk\ell}\sum^{*} Y_{i}Y_{j}Y_{k}Y_{\ell} & =& 3\sum\limits_{ij} {Y_{i}^{2}}{Y_{j}^{2}} - 6\sum\limits_{i}{Y_{i}^{4}} = 3n^{2}{\mu_{2}^{2}}-6n\mu_{4} \end{array} $$

so that

$$\mu_{\emptyset} = \frac1{(n-1)(n-2)(n-3)}\left(3n{\mu_{2}^{2}}-6\mu_{4}\right). $$

We may summarize these results via

$$\left(\begin{array}{l} \mu_{4k}\\ \mu_{3k}\\ \mu_{2p}\\ \mu_{1p}\\ \mu_{\emptyset} \end{array} \right) = A\left(\begin{array}{l} {\mu_{2}^{2}}\\ \mu_{4} \end{array} \right), $$

where the matrix *A* is given in the statement of Lemma 2.

Now

$$\begin{array}{@{}rcl@{}} n^{4}\mathbb{E}\left(\widetilde{\beta}_{g}\widetilde{\beta}_{h}\widetilde{\beta}_{r}\widetilde{\beta}_{s}\right) & =& \sum\limits_{\textit{ijk}\ell} X_{gi}X_{hj}X_{rk}X_{\textit{s}\ell}\mathbb{E}(\widetilde{Y}_{i}\widetilde{Y}_{j}\widetilde {Y}_{k}\widetilde{Y}_{\ell})\\ & = &\mu_{4k}\sum\limits_{i} X_{gi}X_{hi}X_{ri}X_{si}\\ &&+\mu_{3k}\underset{ij}\sum^{*}\left(X_{gi}X_{hi}X_{ri}X_{sj} +X_{gi}X_{hi}X_{rj}X_{si}\right.\\ &&+\left. X_{gi}X_{hj}X_{ri}X_{si} +X_{gj}X_{hi}X_{ri}X_{si} \right)\\ &&+\mu_{2p} \underset{ij}\sum^{*}\left(X_{gi}X_{hi}X_{rj}X_{sj} +X_{gi}X_{hj}X_{ri}X_{sj}\right.\\ &&+\left.X_{gi}X_{hj}X_{rj}X_{si}\right)\\ &&+\mu_{1p} \underset{ijk}\sum^{*}\left(X_{gi}X_{hi}X_{rj}X_{sk} + X_{gi}X_{hj}X_{ri}X_{sk}\right.\\ &&+\left. X_{gi}X_{hj}X_{rk}X_{si} + X_{gi}X_{hj}X_{rj}X_{sk}\right.\\ &&+\left. X_{gi}X_{hj}X_{rk}X_{sj} + X_{gi}X_{hj}X_{rk}X_{sk} \right)\\ &&+\mu_{\emptyset}{\sum\nolimits}^{*} X_{gi}X_{hj}X_{rk}X_{\textit{s}\ell}. \end{array} $$

Next, we write the terms of $n^{4}\mathbb {E}\left (\widetilde {\beta }_{g}\widetilde {\beta }_{h}\widetilde {\beta }_{r}\widetilde {\beta }_{s}\right)$ using $\bar {X}_{\textit {ghrs}}$ and similar moments.

The coefficient of *μ*_4*k*_ is $\sum _{i}X_{\textit {gi}}X_{\textit {hi}}X_{\textit {ri}}X_{\textit {si}} = n\bar X_{\textit {ghrs}}$. The coefficient of *μ*_3*k*_ contains

$$ \begin{aligned} \underset{ij}\sum^{*} X_{gi}X_{hi}X_{ri}X_{sj} &= \sum\limits_{ij} X_{gi}X_{hi}X_{ri}X_{sj} - \sum\limits_{i} X_{gi}X_{hi}X_{ri}X_{si}\\ &= -n\bar{X}_{ghrs} \end{aligned} $$

and after summing all four such terms, the coefficient is $-4n\bar {X}_{\textit {ghrs}}$. The coefficient of *μ*_2*p*_ contains

$$\begin{array}{@{}rcl@{}} \underset{ij}\sum^{*} X_{gi}X_{hi}X_{rj}X_{sj} &=& \sum\limits_{ij} X_{gi}X_{hi}X_{rj}X_{sj} -\sum\limits_{i} X_{gi}X_{hi}X_{ri}X_{si}\\ &=& -n\bar{X}_{ghrs} \end{array} $$

and accounting for all three terms yields $-3n\bar {X}_{\textit {ghrs}}$.

The coefficient of *μ*_1*p*_ contains

$$\begin{array}{@{}rcl@{}} \underset{ijk}\sum^{*} X_{gi}X_{hi}X_{rj}X_{sk} & =& \sum\limits_{ijk}X_{gi}X_{hi}X_{rj}X_{sk} -\sum\limits_{ij}X_{gi}X_{hi}X_{ri}X_{sj} \\ && -\sum\limits_{ik}X_{gi}X_{hi}X_{rj}X_{si} -\sum\limits_{jk}X_{gi}X_{hi}X_{rj}X_{sj}\\ &&+\,2\sum\limits_{i} X_{gi}X_{hi}X_{ri}X_{si} \\ & =& -\,n^{2}\bar{X}_{gh}\bar{X}_{rs} +2n\bar{X}_{ghrs}. \end{array} $$

Summing all 6 terms, we find that the coefficient is

$$-2n^{2}\left(\bar{X}_{gh}\bar{X}_{rs} +\bar{X}_{gr}\bar{X}_{hs} +\bar{X}_{gs}\bar{X}_{hr}\right) +12n\bar{X}_{ghrs}. $$

The coefficient of *μ*_∅_ is, using (6),

$${} {\fontsize{9.4pt}{9.6pt}\selectfont{\begin{aligned} \underset{ijk\ell}\sum^{*} X_{gi}X_{hj}X_{rk}X_{\textit{s}\ell} & = \sum\limits_{ij}\left(X_{gi}X_{hj}X_{ri}X_{sj} +X_{gi}X_{hj}X_{rj}X_{si}\right.\\ &\quad+\left.X_{gi}X_{hi}X_{rj}X_{sj} \right) - 6\sum\limits_{i}X_{gi}X_{hi}X_{ri}X_{si}\\ & = n^{2}\!\left(\bar{X}_{gh}\bar{X}_{rs}\,+\,\bar{X}_{gr}\bar{X}_{hs}+\bar{X}_{gs}\bar{X}_{hr}\right)-6n\bar {X}_{ghrs}. \end{aligned}}} $$

We may summarize these results via

$$\begin{array}{@{}rcl@{}} \mathbb{E}\left(\widetilde{\beta}_{g}\widetilde{\beta}_{h}\widetilde{\beta}_{r}\widetilde{\beta}_{s}\right) &=&\left (\begin{array}{l} \mu_{4k}\\ \mu_{3k}\\ \mu_{2p}\\ \mu_{1p}\\ \mu_{{\emptyset}} \end{array} \right)^{\mathsf{T}} B\left(\begin{array}{l} \bar{X}^{*}_{ghrs}/n^{2}\\[1ex] \bar{X}_{ghrs}/n^{3} \end{array} \right), \quad\text{for}\\ B &=&\left(\begin{array}{rr} 0 & 1\\ 0 & -4\\ 1 & -3\\ -2 & \,12\\ 1 & -6 \end{array} \right), \end{array} $$

where $\bar {X}^{*}_{gh,rs} = \bar {X}_{\textit {gh}}\bar {X}_{\textit {rs}}+\bar {X}_{\textit {gr}}\bar {X}_{\textit {hs}}+\bar {X}_{\textit {gs}}\bar {X}_{\textit {hr}}$, completing the proof of Lemma 2.

These moment expressions have been checked by comparing the variance expression for the quadratic test statistic to that obtained by enumerating all permutations of a small data set. They match.

The expression in Lemma 2 is complicated, but it is simple to compute; we need only two moments of *Y*, two cross-moments of *X*, and the 2×2 matrix *A* ^T^ *B*. The matrix *A* depends on the experiment through *n*. Using Lemma 2 we can obtain the covariance between ${\widetilde {\beta }^{2}_{g}}$ and ${\widetilde {\beta }^{2}_{h}}$.

#### **Corollary2**.

For an experiment with *n*≥4, and genes *g*,*h*,

$$\text{cov}\left({\widetilde{\beta}^{2}_{g}},{\widetilde{\beta}^{2}_{h}}\right) \,=\,\left(\! \begin{array}{l} {\mu_{2}^{2}}\\ \mu_{4} \end{array}\! \right)^{\mathsf{T}} A^{\mathsf{T}} B \left(\! \begin{array}{l} \bar{X}^{*}_{gghh} /n^{2}\\[1ex] \bar{X}_{gghh}/n^{3} \end{array}\! \right) -\frac{{\mu_{2}^{2}}}{(n-1)^{2}}\bar{X}_{gg}\bar{X}_{hh}, $$

where $\bar {X}^{*}_{\textit {gghh}} = \bar {X}_{\textit {gg}}\bar {X}_{\textit {hh}} + 2\bar {X}_{\textit {gh}}^{2}$ with *A* and *B* as given in Lemma 2.

#### *Proof*.

The covariance is $\mathbb {E}\left ({\widetilde {\beta }^{2}_{g}}{\widetilde {\beta }^{2}_{h}}\right)-\mathbb {E}\left ({\widetilde {\beta }^{2}_{g}}\right) \mathbb {E}\left ({\widetilde {\beta }^{2}_{h}}\right)$. Applying Lemma 2 to the first expectation and Lemma 1 to the other two yields the result.

### Rotation moments of test statistics

Rotation sampling [35,36] provides an alternative to permutations, and is justified if either *X* or *Y* has a Gaussian distribution. It is simple to describe when $Y\sim \mathcal {N}(\mu,\sigma ^{2}I_{n})$, and simplifies further in the special case *μ*=0. In the latter case we can replace *Y* by $\widetilde {Y} = QY$ where $Q\in \mathbb {R}^{n\times n}$ is a random orthogonal matrix (independent of both *X* and *Y*), and the distribution of our test statistics is unchanged under the null hypothesis that *X* and *Y* are independent.

Rotation tests work by repeatedly sampling from the uniform distribution on random orthogonal matrices and recomputing the test statistics using $\widetilde {Y}$ instead of *Y*. They suffer from resampling granularity but not data granularity because *Q* has a continuous distribution (for *n*≥2).

To take account of centering we need to use a rotation test appropriate for $Y\sim \mathcal {N}(\mu,\sigma ^{2} I_{n})$. Langsrud [36] does this by choosing rotation matrices that leave the population mean fixed. He rotates the data in an *n*−1 dimensional space orthogonal to the vector 1_*n*_. To get such a rotation matrix, he first selects an orthogonal contrast matrix $W\in \mathbb {R}^{n\times (n-1)}$. This matrix satisfies *W* ^T^ *W*=*I*_*n*−1_ and *W* ^T^1_*n*_=0_*n*−1_. Then he generates a uniform random rotation $Q^{*}\in \mathbb {R}^{(n-1)\times (n-1)}$ and delivers $\widetilde {Y}=QY$, where $Q = \frac 1n1_{n}1_{n}^{\mathsf {T}} +WQ^{*}W^{\mathsf {T}}$. More generally if $Y\sim \mathcal {N}(Z\gamma,\sigma ^{2} I_{n})$, for a linear model *Z* *γ*, Langsrud [36] shows how to rotate *Y* in the residual space of this model, leaving the fits unchanged.

Wu *et al.* [9] have implemented rotation sampling for microarray experiments in their method, ROAST. They speed up the sampling by generating a random vector instead of a random matrix. For some tests, permutations and rotations have the same moments, and so our approximations are approximations of rotation tests as much as of permutation tests.

Our rotation method approximation performs very similarly to the permutation method. We let $\widetilde {Y} = QY$ for $Q = (\frac 1n1_{n}1_{n}^{\mathsf {T}} + WQ^{*}W^{\mathsf {T}})$ where *Q* ^∗^ is a uniform random *n*−1×*n*−1 rotation matrix and the contrast matrix $W\in \mathbb {R}^{n\times (n-1)}$ satisfies *W* ^T^1_*n*_=0_*n*−1_ and *W* ^T^ *W*=*I*_*n*−1_ and then $\widetilde {\beta }$, $\widetilde {T}$ and $\widetilde {C}$ are defined as for permutations, substituting $\widetilde {Y}$ for *Y*.

The variance of the quadratic test statistic depends on *which* contrast matrix *W* one chooses, and so it cannot always match the permutation variance. This difference disappears asymptotically as *n*→*∞*. Our main results on rotation sampling are that the other moments match, as follows.

#### **Lemma3**.

For an experiment with *n*≥2 including genes *g* and *h*, the moments $\mathbb {E}\left (\widetilde {\beta }_{g}\right)$ and $\mathbb {E}\left (\widetilde {\beta }_{g}\widetilde {\beta }_{h}\right)$ are identical to their permutation counterparts, regardless of the choice for *W*.

We prove Lemma 3 below. It has the following immediate consequence.

#### **Corollary3**.

For an experiment with *n*≥2, $\mathbb {E}\left (\widetilde {T}_{G,w}\right)$, $\text {var}\left (\widetilde {T}_{G,w}\right)$ and $\mathbb {E}\left (\widetilde {C}_{G,w}\right)$ are the same whether $\widetilde {Y}$ is formed by permutation or rotation of *Y*.

#### *Proof of Lemma*3.

We begin with some low order moments of orthogonal random matrices. For integers *n*≥*k*≥1, let $V_{n,k} = \left \{ Q\in \mathbb {R}^{n\times k}\mid Q^{\mathsf {T}} Q = I_{k}\right \}$, known as the Stiefel manifold. We will make use of the uniform distributions on *V*_*n*,*k*_. There is a natural identification of *V*_*n*,1_ with the unit sphere.

Let $Q\in \mathbb {R}^{n\times n}$ be a uniform random rotation matrix. This implies, among other things, that each column of *Q* is a uniform random point on the unit sphere in *n* dimensions.

By symmetry, we find that $\mathbb {E}(Q_{\textit {ij}}) = 0$. Similarly $\mathbb {E}(Q_{\textit {ij}}^{2}) = \mathbb {E}((1/n)\sum _{j=1}^{n} Q_{\textit {ij}}^{2})=1/n$ and $\mathbb {E}(Q_{\textit {ij}}Q_{\textit {rs}}) =0$ unless *i*=*r* and *j*=*s*. Let $X_{i}\in \mathbb {R}^{p}$ where *p*=|*G*| and $Y_{i}\in \mathbb {R}$ for *i*=1,…,*n*. Both *X*_*i*_ and *Y*_*i*_ are centered: $\sum _{i}X_{i}=0$ and $\sum _{i} Y_{i}=0$.

The sample coefficients for genes *g*∈*G* are given by the vector $\hat \beta = (1/n)\sum _{i}X_{i}Y_{i}\in \mathbb {R}^{|G|}$. The reference distribution is formed by sampling values of $\widetilde {\beta } = (1/n)\sum _{i}X_{i}\widetilde {Y}_{i}$ where $\widetilde {Y}$ is a rotated version of *Y*.

The rotation is one that preserves the mean of *Y* while rotating in the *n*−1 dimensional space of contrasts. As in [36], we let $W\in \mathbb {R}^{n\times (n-1)}$ be any fixed contrast matrix satisfying *W* ^T^ *W*=*I*_*n*−1_ and *W* ^T^1_*n*_=0_*n*−1_. Then the rotated version of *Y* is

$$\widetilde{Y} = W Q W^{\mathsf{T}} Y,\quad\text{where}\quad Q\sim\mathsf{U}(V_{n-1,n-1})$$

is a uniform random *n*−1 dimensional rotation matrix.

It is convenient to introduce centered quantities $X^{c} = W^{\mathsf {T}} X\in \mathbb {R}^{(n-1)\times p}$, $Y^{c} = W^{\mathsf {T}} Y\in \mathbb {R}^{n-1}$ and $\widetilde {Y}^{c} = W^{\mathsf {T}} \widetilde {Y}\in \mathbb {R}^{n-1}$. These sum to zero even when *X*, *Y* and $\widetilde {Y}$ do not. Their main difference from those variables is that they have *n*−1 rows, not *n*.

Now $\widetilde {\beta }= (1/n)X^{\mathsf {T}} \widetilde {Y}=(1/n) X^{\mathsf {T}} WQW^{\mathsf {T}} Y=(1/n){X^{c}}^{\mathsf {T}} Q Y^{c}$, so

$$\mathbb{E}\left(\widetilde{\beta}\right) = (1/n){X^{c}}^{\mathsf{T}}\mathbb{E}(Q){Y^{c}}^{\mathsf{T}} = 0, $$

matching the moment under permutation. For the rest of the proof, we need the covariance matrix of $\widetilde {\beta }$. Now

$$ \mathbb{E}\left(\widetilde{\beta}\widetilde{\beta}^{\mathsf{T}} \right) \,=\,\frac1{n^{2}}{X^{c}}^{\mathsf{T}} \mathbb{E}\left(\! Q^{\mathsf{T}} Y^{c}{Y^{c}}^{\mathsf{T}}Q \!\right){X^{c}}^{\mathsf{T}}\,=\, \frac1{n^{2}} {X^{c}}^{\mathsf{T}}\mathbb{E}\left(Q^{\mathsf{T}} Z Q\right)X^{c}\notag $$

where $Z = Y^{c}{Y^{c}}^{\mathsf {T}} \in \mathbb {R}^{(n-1)\times (n-1)}$.

The *ij* element of *Q* ^T^ *Z* *Q* is $(Q^{\mathsf {T}} ZQ)_{\textit {ij}} = \sum _{k=1}^{n-1} \sum _{\ell =1}^{n-1} Z_{\textit {k}\ell } Q_{\textit {ki}} Q_{\ell j}$ which has expected value

$$ \sum\limits_{k=1}^{n-1} \sum\limits_{\ell=1}^{n-1} Z_{\textit{k}\ell} 1_{k=\ell}1_{i=j}/(n-1) \,=\,\frac{1_{i=j}}{n-1}\sum\limits_{k=1}^{n-1}Z_{kk} \,=\, 1_{i=j}\frac{n}{n-1}\mu_{2} $$

where $\mu _{2} = (1/n)\sum _{i=1}^{n}{Y_{i}^{2}} = (1/n)\sum _{i=1}^{n}{{Y^{c}_{i}}}^{2}$. That is

$$\mathbb{E}\left(Q^{\mathsf{T}} ZQ\right) = \frac{n\mu_{2}}{n-1}I_{n-1} $$

and so

$$\mathbb{E}\left(\widetilde{\beta}\widetilde{\beta}^{\mathsf{T}}\right) = \frac{\mu_{2}}{n(n-1)}{X^{c}}^{\mathsf{T}} X^{c}. $$

In particular $\mathbb {E}\left (\widetilde {\beta }_{g}\widetilde {\beta }_{h}\right) = \mathbb {E}\left (\widetilde {\beta }\widetilde {\beta }^{\mathsf {T}}\right)_{\textit {gh}} = \bar {X}_{\textit {gh}}\mu _{2}/(n-1)$, matching the value under permutation.

### Fourth moments

Here we show that the variance of $\widetilde {C}_{G,w}$ in rotation sampling can depend on the specific matrix *W* used. We need fourth moments like $\mathbb {E}\left ({\widetilde {\beta }_{r}^{2}}{\widetilde {\beta }_{s}^{2}}\right)$. Those in turn depend on fourth moments of *Q*.

Anderson, Olkin and Underhill [37] give

(7) $$ \mathbb{E}\left(Q_{ij}^{4}\right) = \frac3{n(n+2)}.  $$

We are interested in all fourth moments $\mathbb {E}(Q_{\textit {ij}}Q_{\textit {k}\ell } Q_{\textit {rs}}Q_{\textit {tu}})$ of *Q*. If any of *j*,*ℓ*,*s*,*u* appears exactly once then the fourth moment is 0 by symmetry. To see this, suppose that index *ℓ* appears exactly once. Now define the matrix $\widetilde {Q}$ with elements

$$\widetilde{Q}_{ij} = \left\{ \begin{array}{lll} -Q_{ij} & j=\ell,\\ Q_{ij} & j\ne \ell. \end{array} \right. $$

If *Q*∼U(*V*_*n*,*n*_) then $\widetilde {Q}\sim \mathsf {U}(V_{n,n})$ too by invariance of U(*V*_*n*,*n*_) to multiplication on the right by the orthogonal matrix diag(1,1,…,1,−1,1,…,1), with a −1 in the *j* ^′^th position. Then

$$\begin{array}{@{}rcl@{}} \mathbb{E}(Q_{ij}Q_{\textit{k}\ell}Q_{rs}Q_{tu}) &=& \!\frac{1}{2}\mathbb{E}\left(Q_{ij}Q_{\textit{k}\ell}Q_{rs}Q_{tu} + \widetilde{Q}_{ij}\widetilde{Q}_{\textit{k}\ell}\widetilde Q_{rs}\widetilde{Q}_{tu}\right)\\ &=&\! \frac{1}{2}\mathbb{E}\!\left(\!Q_{ij}Q_{\textit{k}\ell}Q_{rs}Q_{tu}\! +\! Q_{ij}(-Q_{\textit{k}\ell})Q_{rs}Q_{tu}\right)\\ &=&0. \end{array} $$

Similarly, because *Q* ^T^ is also uniformly distributed on *V*_*n*,*n*_ we find that if any of *i*,*k*,*r*,*t* appear exactly once the moment is zero. If one index appears exactly three times, then some other moment must appear exactly once. As a result, the only nonzero fourth moments are products of squares and pure fourth moments. Their values are given in the Lemma below.

#### **Lemma4**.

Let *Q*∼U(*V*_*n*,*n*_). Then

$$\mathbb{E}\left(Q_{ij}^{2}Q_{rs}^{2} \right) =\left\{ \begin{array}{lll} \frac{3}{n(n+2)}, & i=r\ \&\ j=s\\[2ex] \frac{1}{n(n+2)}, & 1_{i=r} + 1_{j=s} = 1\\[2ex] \frac{n+1}{n(n-1)(n+2)}, & i\ne r\ \&\ j\ne s. \end{array} \right. $$

#### *Proof*.

The first case was given by [37]. For the second case, there is no loss of generality in computing $\mathbb {E}\left (Q_{11}^{2}Q_{21}^{2} \right)$. The vector (*Q*_11_,*Q*_21_,…,*Q*_*n*1_) is uniformly distributed on the sphere. Given *Q*_11_, the point (*Q*_21_,*Q*_31_,…,*Q*_*n*1_) is uniformly distributed on the *n*−1 dimensional sphere of radius $\sqrt {1-Q_{11}^{2}}$. Therefore $\mathbb {E}\left (Q_{21}^{2} \mid Q_{11} \right) = \left (1-Q_{11}^{2}\right)/(n-1)$ and so

$$\begin{array}{@{}rcl@{}} \mathbb{E}\left(Q_{11}^{2}Q_{21}^{2}\right) &=& \frac1{n-1}\mathbb{E}\left(Q_{11}^{2}-Q_{11}^{4}\right)\\ &=&\frac{1}{n-1}\left(\frac1n - \frac3{n(n+2)}\right) =\frac{1}{n(n+2)}. \end{array} $$

For the remaining case we let $\theta = \mathbb {E}(Q_{\textit {ij}}^{2}Q_{\textit {rs}}^{2})$ for *i*≠*r* and *j*≠*s*. Summing over *n* ^4^ combinations of indices we find that

$$\sum\limits_{i=1}^{n}\sum\limits_{j=1}^{n}\sum\limits_{r=1}^{n}\sum\limits_{s=1}^{n}Q_{ij}^{2}Q_{rs}^{2} =\left(\sum\limits_{ij}Q_{ij}^{2}\right)^{2} = n^{2} $$

by orthogonality of *Q*. Therefore

$$ \begin{aligned} n^{2} &= \mathbb{E}\left(\sum\limits_{ij}\sum\limits_{rs}Q_{ij}^{2}Q_{rs}^{2} \right)\\ &= n^{2} \mathbb{E}\left(Q_{11}^{4} \right) + 2n^{2}(n-1)\mathbb{E}(Q_{11}^{2}Q_{12}^{2}) +n^{2}(n-1)^{2}\theta. \end{aligned} $$

Solving for *θ* we get

$$ \begin{array}{lcrr}\theta =\frac{n^2-\frac{3n}{n+2}-\frac{2n\left(n-1\right)}{n+2}}{n^2{\left(n-1\right)}^2}=\frac{n+1}{n\left(n-1\right)\left(n+2\right)}.\kern2em & & & \end{array} $$

The exact value of $\mathbb {E}\left ({\widetilde {\beta }^{2}_{r}}{\widetilde {\beta }^{2}_{s}}\right)$ is a very bulky expression. It does however include a term with a nonzero coefficient multiplied by $\sum _{i=1}^{n} ({Y^{c}_{i}})^{4}$ times a similar quantity involving *X*. This fourth moment depends on the matrix *W* used. To see this in an example consider that for *n*=3, we could take

$$W^{\mathsf{T}} =\left(\begin{array}{lll} \frac1{\sqrt{2}} & -\frac1{\sqrt{2}} &\quad 0\\ \frac1{\sqrt{6}} & \frac1{\sqrt{6}} & -\frac{{2}}{\sqrt{6}} \end{array} \right) $$

Then $\sum _{i} (W^{\mathsf {T}} Y)_{i}^{4} = (5/9){Y_{1}^{4}}+(5/9){Y_{2}^{4}}+(1/9){Y_{3}^{4}}$. Permuting the columns of *W* ^T^ would then change which *Y*_*i*_ got the small coefficient. Lemma 4 convinces us that the effect of *W* on ROAST vanishes for $\text {var}(\widetilde {C}_{G,w})$ as *n* increases. That Lemma shows that the cross moments $\mathbb {E}\left (Q_{\textit {ij}}^{2}Q_{\textit {rs}}^{2}\right)$ for *i*≠*r* or *j*≠*s*, are of the same order of magnitude as $\mathbb {E}(Q_{\textit {ij}}^{4})$. Those moments appear in coefficients of only second moments of *W* ^T^ *Y* and *X* ^T^ *Y*. Also there are many more of them so they dominate the cross moments $\mathbb {E}\left ({\widetilde {\beta }_{r}^{2}}{\widetilde {\beta }_{s}^{2}}\right)$.

### Computation and costs

To facilitate computation for the linear statistic, we reduce each gene set to a single pseudo-gene $X_{\textit {Gi}} = \sum \limits _{g\in G}w_{g}X_{\textit {gi}}$ and then let

$$\bar X_{G} = \frac1{n}\sum\limits_{i=1}^{n} X_{Gi}\quad\text{and}\quad \bar X_{GG} = \frac1{n}\sum\limits_{i=1}^{n} X_{Gi}^{2}.$$

The weights *w* have been absorbed into the pseudo-gene to simplify notation. We define

$$\begin{array}{@{}rcl@{}} \hat\beta_{G} &=& \sum\limits_{g\in G}w_{g} \hat\beta_{g} = \frac1n\sum\limits_{i}X_{Gi}Y_{i},\quad\text{and}\\[-4pt] \widetilde\beta_{G} &=& \sum_{g\in G}w_{g} \widetilde\beta_{g} = \frac1n\sum\limits_{i}X_{Gi}\widetilde Y_{i}. \end{array} $$

Our permuted linear test statistic is $\widetilde T_{G,w} = \widetilde \beta _{G}$, with

(8) $$\begin{array}{@{}rcl@{}} \text{var}\left(\widetilde T_{G,w}\right) &= \text{var}\left(\widetilde\beta_{G}\right) = \frac{\mu_{2}}{n-1}\bar X_{GG}. \end{array} $$

For the beta approximation, we need the range of $\widetilde {T}_{G,w}$. Let the sorted *Y* values be *Y*_(1)_≤*Y*_(2)_≤…≤*Y*_(*n*)_ and the sorted *X*_*Gi*_ values be *X*_*G*(1)_≤*X*_*G*(2)_≤…≤*X*_*G*(*n*)_. Then the range of $\widetilde {T}_{G,w}$ is [ *A*,*B*], where

$$\begin{array}{@{}rcl@{}} A & = \frac1n \sum\limits_{i=1}^{n} X_{G(i)}Y_{(n+1-i)},\quad\text{and}\quad B = \frac1n \sum\limits_{i=1}^{n} X_{G(i)}Y_{(i)}. \end{array} $$

For a *σ* *t*_(*ν*)_ reference distribution we would also need $\mathbb {E}\left (\!\widetilde {T}_{G,w}^{4}\!\right)=\mathbb {E}\left ({\widetilde {\beta }_{G}^{4}}\right)$. We can apply Lemma 2 to the pseudo-gene resulting in

(9) $$ \mathbb{E}({\widetilde{\beta}_{G}^{4}}) =\left(\begin{array}{l} {\mu_{2}^{2}}\\ \mu_{4} \end{array} \right) A^{\mathsf{T}} B \left(\begin{array}{l} 3\bar{X}_{GG}^{2}/n^{2}\\ \bar{X}_{GGGG}/n^{3} \end{array} \right),  $$

where $\bar {X}_{\textit {GGGG}} = \frac 1{n}\sum _{i=1}^{n} X_{\textit {Gi}}^{4}$.

We considered using a *σ* *t*_(*ν*)_ reference distribution for $\widetilde T_{G,w}$, taking into account the fourth moment of $\widetilde {T}_{G,w}$ (9). We have often (in fact usually) found that $\mathbb {E}\left (\widetilde T_{G,w}^{4}\right)<3\mathbb {E}\left (\widetilde T_{G,w}^{2}\right)^{2}$; that is, lighter tails than the normal. This implies a negative kurtosis for the permutation distribution, and *t* distributions have positive kurtosis. For this reason we use a beta approximation and not a *t* approximation.

For the quadratic statistic we have found it useful to replace *X*_*gi*_ by $\sqrt {w_{g}}X_{\textit {gi}}$ in precomputation. That step is only valid for non-negative *w*_*g*_, but those are the ones of most interest. Note that mixing positive and negative *w*_*g*_’s would lead to a test statistic where evidence that gene *g* is non-null could cancel out the evidence of gene *h* being non-null for *g*,*h*∈*G*. Then we use formulas for $\mathbb {E}\left (\widetilde C_{G,w}\right)$ and $\text {var}\left (\widetilde C_{G,w}\right)$ with all *w*_*g*_=*w*_*h*_=1 (5).

Now we consider the computational cost. The cost to compute all of the *X*_*Gi*_ is dominated by *np* multiplications. It then takes *n* more multiplications to get $\hat \beta _{g}$ and another *n* to get $\bar X_{\textit {GGe}}$. It costs *n* multiplications to get *μ*_2_ and *μ*_4_. That step can be done once and can be used for all gene sets. The cost for the Gaussian approximation $\mathcal {N}\left (0,\text {var}(\widetilde T_{G,w})\right)$ is dominated by *n*(*p*+2) multiplications.

For the beta approximation there is also a cost proportional to *n* log(*n*) in the sorting to compute limits *A* and *B*. That adds a cost comparable to a multiple of log(*n*) permutations. We judge that the cost of sorting is usually minor for *n* and *p* of interest in bioinformatics.

A permutation analysis requires *nM* multiplications, after computing *X*_*Gi*_, for a total of *n*(*M*+*p*). It is very common for *p* to be a few tens and *M* to be many thousands or more. Then we can simplify the costs to *n*(*M*+*p*)≈*n* *M* and *n*(2+*p*)≈*n* *p*. The moment method costs about as much as doing *p* permutations. When the gene set has tens of genes and the permutation method uses many thousands or even several million permutations, the computational cost is quite large.

The pseudo-gene technique is more expensive for the quadratic statistics. The dominant cost in computing $\widehat C_{G,w}$ is still the *np* multiplications required to compute $\widehat \beta _{g}$ for *g*∈*G*. We can also compute $\mathbb {E}(\widetilde C_{G,w})$ in about this amount of work.

The cost of computing $\text {var}(\widetilde C_{G,w})$ by a straightforward algorithm is at least *n* *p* ^2^, because we need $\bar X_{\textit {gh}}$ and $\bar X_{\textit {gghh}}$ for all *g*,*h*∈*G*. Some parts of that computation can be sped up to *O*(*n* *p*) by rewriting the expression as described below. One of the terms however does not reduce to *O*(*n* *p*). A straightforward implementation costs *O*(*n* *p* ^2^) while an alternative expression costs *O*(*n* ^2^ *p*). The latter is valuable in settings where the gene sets are large compared to the sample size. In the former case, the moment approximation has cost comparable to *O*(*p* ^2^) permutations. If *n*<*p* then the latter case is like *np* permutations, so the quadratic cost is comparable to on the order of *p*∗ min(*n*,*p*) permutations.

Recall from Corollary 2 that in an experiment with *n*≥4 and genes *g*,*h*,

$$ \text{cov}\left({\widetilde\beta^{2}_{g}},{\widetilde\beta^{2}_{h}}\right) \,=\,\left(\! \begin{array}{l} {\mu_{2}^{2}}\\ \mu_{4} \end{array}\! \right)^{\mathsf{T}} A^{\mathsf{T}} B \left(\! \begin{array}{l} \bar{X}^{*}_{gghh} /n^{2}\\[1ex] \bar{X}_{gghh}/n^{3} \end{array}\! \right) -\frac{{\mu_{2}^{2}}}{(n-1)^{2}}\bar X_{gg}\bar X_{hh}, $$

where $\bar X^{*}_{\textit {gghh}} = \bar X_{\textit {gg}}\bar X_{\textit {hh}} + 2\bar X_{\textit {gh}}^{2}$ and *A* ^T^ *B* is a given 2×2 matrix.

To compute

$$\text{var}\left(\widetilde C_{G,w}\right) = \sum\limits_{g\in G}\sum\limits_{h\in G} w_{g}w_{h}\text{cov}\left(\widetilde{\beta}_{g}^{2},{\widetilde{\beta}_{h}^{2}}\right) $$

we need *μ*_2_, *μ*_4_ and *A* ^T^ *B* which are very inexpensive. We also need

$$S_{1}\equiv \sum\limits_{g\in G}\sum\limits_{h\in G}w_{g}w_{h}\bar X_{gg}\bar X_{hh} = \left(\, \sum\limits_{g\in G}w_{g}\bar X_{gg}\right)^{2}. $$

By expressing *S*_1_ as a square, we find that it can be computed in *O*(*n* *p*) work, not *O*(*n* *p* ^2^) which a naive implementation would provide. We can compute all of the $\bar X_{\textit {gg}}$’s in *np* multiplications and this is the largest part of the cost. If gene *g* belongs to many gene sets *G* we only need to compute $\bar X_{\textit {gg}}$ once and so the cost per additional gene set could be lower.

A similar analysis yields that

$$S_{2}\equiv\sum_{g\in G}\sum_{h\in G}w_{g}w_{h}\bar X_{gghh} = \frac1n\sum_{i=1}^{n} \Biggl(\, \sum_{g\in G}w_{g}X_{gi}^{2}\Biggr)^{2} $$

is also an *O*(*n* *p*) computation. Unfortunately $S_{3} \equiv \sum _{g\in G}\sum _{h\in G}\bar X_{\textit {gh}}^{2}$ does not reduce to an *O*(*n* *p*) computation. As written it costs *O*(*n* *p* ^2^). In cases where *p*>*n*, we can however reduce the cost to *O*(*n* ^2^ *p*) via

$$\begin{array}{@{}rcl@{}} S_{3} & =& \sum\limits_{g\in G}\sum\limits_{h\in G}w_{g}w_{h}\left(\,\frac1{n}\sum_{i=1}^{n}X_{gi}X_{hi}\right)^{2}\\ &=& \frac1{n^{2}}\sum\limits_{g\in G}\sum\limits_{h\in G}w_{g}w_{h} \sum\limits_{i=1}^{n} X_{gi}X_{hj}\sum\limits_{j=1}^{n}X_{gj}X_{hj}\\ & =& \frac1{n^{2}} \sum_{i=1}^{n}\sum\limits_{j=1}^{n} \left(\,\sum\limits_{g\in G}w_{g}X_{gi}\right)^{2}. \end{array} $$

In terms of these sum quantities,

$$ \text{var}(\widetilde C_{G,w}) =\left(\begin{array}{l} {\mu_{2}^{2}}\\ \mu_{4} \end{array} \right)^{\mathsf{T}} A^{\mathsf{T}} B \left(\begin{array}{c} (S_{1} + 2S_{3})/n^{3}\\ S_{2}/n^{3} \end{array} \right) -\frac{{\mu_{2}^{2}}}{(n-1)^{2}}S_{1}. $$

## Additional file

Additional file 1
**Table S1.** A table of the moment-based *p*-values for 6,303 gene sets in three genome-wide expression studies.

## Abbreviations

GEV: Generalized extreme value
GO: Gene Ontology
GSEA: Gene set enrichment analysis
PD: Parkinson’s disease
